# Analysis of structural injury patterns in peripapillary retinal nerve fibre layer and retinal ganglion cell layer in ethambutol-induced optic neuropathy

**DOI:** 10.1186/s12886-021-01881-y

**Published:** 2021-03-10

**Authors:** Wen-Yan Sheng, Ling-Ya Su, Wei Ge, Shuang-Qing Wu, Li-Wei Zhu

**Affiliations:** Department of Ophthalmology, Zhejiang Hospital of Intergrated Traditional Chinese and Western Medicine, 208 Huancheng Road East, Zhejiang 310003 Hangzhou, People’s Republic of China

**Keywords:** Cirrus-HD optical coherence tomography, Retinal nerve fibre layer, Ethambutol, Ganglion cell inner plexiform layer, Toxic optic neuropathy

## Abstract

**Background:**

We investigated structural injury patterns in the peripapillary retinal nerve fibre layer (p-RNFL) and ganglion cell inner plexiform layer (GCIPL) caused by ethambutol treatment.

**Methods:**

Sixty-four patients undergoing ethambutol treatment at Zhejiang Chinese Medicine and Western Medicine Integrated Hospital were recruited. Fourteen (14) exhibited visual dysfunction (abnormal group), and the remaining 50 had no visual dysfunction (subclinical group). The thickness of the p-RNFL, total macular retina layer and GCIPL were measured using Cirrus-HD Optical coherence tomography (Cirrus-HD OCT, Cirrus high-definition optical coherence tomography), and compared with 60 healthy, age-matched controls.

**Results:**

The p-RNFL thickness was similar in both subclinical and control groups. When compared with the control group, p-RNFL thickness in the abnormal group was significantly increased in the inferior and superior quadrants (GEE, *P* = 0.040, *P* = 0.010 respectively). In contrast with the subclinical group, p-RNFL thickness in the inferior quadrant was increased in the abnormal group (GEE, *P* = 0.047). The GCIPL thickness in the inferonasal and inferior sectors was significantly deceased in the subclinical group when compared with controls (GEE, *P* = 0.028, *P* = 0.047, respectively). The average and minimum value of GCIPL thickness, and thickness in the superonasal, inferior, inferotemporal, superotemporal and superior sectors were significantly decreased in the abnormal group when compared with controls (GEE, *P* = 0.016, *P* = 0.001, *P* = 0.028, *P* = 0.010, *P* = 0.012, *P* = 0.015, *P* = 0.010, respectively). The cube average macular thickness (CAMT) in the abnormal group was significantly thinner than controls (GEE, *P* = 0.027).

**Conclusions:**

GCIPL measurements using Cirrus-HD OCT detected retinal ganglion cell layer loss following ethambutol treatment, before visual dysfunction occurred.

## Background

Ethambutol is a first-line antibacterial drug used to treat *Mycobacterium tuberculosis* infection, however, the main side effect is optic neurotoxicity [1]. It was previously reported that 1–2% of patients taking ethambutol doses at 15–25 mg/kg/d could develop ethambutol-induced optic neuropathy (EON) [1, 2]. Globally, every year, approximately 100,000 people experience EON [3]. Typical manifestations include; symmetrical, painless, progressive vision loss, accompanied by abnormal colour vision and visual field defect. EON may be reversible at early stages, but a delayed diagnosis results in permanent vision loss [2, 3]. Patients with tuberculosis taking ethambutol should undergo regular visual acuity, colour vision and visual field monitoring. However, early detection of EON is difficult to confirm by these subjective examinations. When a patient experiences visual dysfunction, the lesions are often irrecoverable [4]. Therefore, a quantitative objective marker for early detection of EON is required.

Optical coherence tomography (OCT) is a non-invasive imaging technique, providing high resolution for retinal micro-structures. It was previously used to measure the thickness of the peripheral retinal nerve fibre layer (p-RNFL) in EON patients [5–9]. In the past, it was postulated that ethambutol exhibited neurophilic characteristics, and that EON was primarily manifested as a retrobulbar optic neuropathy [4]. Recently however, ganglion cell inner plexiform layer (GCIPL) thickness was decreased in EON patients when measured by OCT, before p-RNFL thickness had decreased, suggesting ethambutol may cause direct damage to retinal ganglion cell layers [10].

Currently, some studies [9, 10] have investigated retinal ganglion cells in patients without significant visual impairment after taking ethambutol, and similarly, retinal ganglion cell injury patterns during early EON stages. Therefore, we investigated the thickness of p-RNFL, GCIPL and the total macular retina layer in patients with tuberculosis taking ethambutol, to determine retinal ganglion cell layer damage and characteristics.

## Methods

### Study subjects

This was a retrospective, cross-sectional, observational study. Sixty-four TB patients who complained of ocular discomfort after taking ethambutol at the Zhejiang Chinese Medicine and Western Medicine Integrated Hospital between January 2018 and June 2019, were recruited. We diagnosed EON based on the following criteria: 1) visual symptoms appeared after ethambutol therapy commencement, and 2) an EON diagnosis had to agree with both major criteria, or one major criterion and two minor criteria (below). Major criteria: 1) abnormal results from colour vision tests and no other reasonable cause for abnormal colour vision, and 2) central or paracentral scotoma on the Goldmann or Humphrey perimeter. Minor criteria: 1) visual field defects other than central or paracentral scotomas, and 2) optic disc pallor [2, 3]. Subjects were included in the abnormal group if visual field defects were observed, accompanied by a decline in visual acuity or colour vision after ethambutol. Visual field defect patterns included cecocentral scotoma, central scotoma, peripheral constriction, altitudinal defect, and hemianopsia. Fifty subjects complaining of ocular discomfort, without any decline in visual acuity, colour vision, or visual field after ethambutol, were included in the subclinical group. Sixty age-matched healthy individuals, without ethambutol treatment, were used as controls.

Exclusion criteria included; 1) intraocular pressure > 21 mmHg (1 mmHg = 0.133 kPa), complicated by other ocular diseases affecting the visual field and optic nerve or visual pathway; 2) refractive error > ± 4.00 diopter sphere and astigmatism of ±2.00 diopters; 3) a history of intraocular surgery; 4) and central nervous system disorders.

This study was approved by the Ethics Committee of Zhejiang Province Hospital of Integrated Traditional and Western Medicine, and was conducted according to the tenets of the Declaration of Helsinki, in its currently applicable version. All subjects signed an informed consent form before participating. Patients discontinued ethambutol when an abnormal visual function was identified.

### General examinations

All subjects underwent ophthalmologic examinations, including best-corrected visual acuity (BCVA), intraocular pressure, colour vision test, slit-lamp microscopy and fundus examination. BCVA was assessed by a Snellen eye chart (decimal acuity) and converted to LogMAR values [11]. Finger counting, hand motion and light perception were converted to LogMAR equivalents of 2.0, 3.0 and 4.0, respectively [12]. Values < 0.1 by LogMar were considered as apparent decreases. An 11-plate Ishihara Colour Test was used for colour vision examination.

### OCT examinations

All subjects were examined using Cirrus-HD OCT (Carl Zeiss Meditec Inc., Dublin, CA, USA) without pupil dilation. All scans were acquired by experienced operators. The p-RNFL was detected using a 3.4 mm circular scan around the optic disc, and the average thickness, and that of four quadrant areas (i.e. superior, inferior, nasal and temporal) were measured.

The total macular layer and volume was examined using the Macular Cube model (512 × 128), and macular measurements included: (1) cube average macular thickness (CAMT), within the diameter of a 6 mm circle in the macula; (2) cube macular volume (CMV), macular volume within the diameter of a 6 mm circle; (3) centre subfield thickness (CST), nine sectors of macular thickness according to 1-, 3- and 6 mm Early Treatment Diabetic Retinopathy Study map, among them the central subfield macular thickness, was the average macular fovea thickness within the diameter of a 1 mm circle.

The GCIPL thickness was assessed using the Cirrus HD-OCT ganglion cell analysis programme, taking the macular fovea as the midpoint, scanning the elliptical area of 4.8 mm in length, a short diameter of 4 mm and an area of 14.13 mm^2^, which was 1 mm from the macular fovea, but within 4 mm. Using this strategy, measurement results were divided into 360° for analysis. The minimum data in this 360° analysis were taken as the ‘minimum GCIPL thickness’. In addition, the average GCIPL thickness was also obtained, and the GCIPL thickness of the superotemporal, superior, superonasal, inferonasal, inferotemporal and inferior areas; each 60° in area.

### Visual field testing

The Humphrey Field Analyzer (Carl Zeiss Meditec) using the SITA-FAST strategy and C30–2 programme, was used for visual field examinations. The mean deviation (MD) in decibels was analysed. A visual field defect was defined as a cecocentral scotoma, central scotoma, peripheral constriction, altitudinal defect, or hemianopsia, etc.

### Statistical analysis

Statistical analyses were conducted using SPSS 19.0 software. The chi-squared test was used for sex distribution. LogMAR and MD values were expressed as medians (interquartile distance) and analysed using nonparametric tests. OCT measurements between the three groups were expressed as the mean ± standard deviation. A generalised estimating equation (GEE) was used to compare the thickness of p-RNFL, GCIPL and the total macular layer between groups, accounting for age, ethambutol treatment duration and within-patient inter-eye correlations. A *P* value < 0.05 was considered statistically significant.

## Results

We enrolled 126 eyes from 64 patients with eye complaints after ethambutol treatment, and 118 eyes from 60 healthy individuals. The eye complains of the 64 patients at the first visit were categorised; gradually decreased visual acuity in 9 (14.1%) patients, blurry vision in 21 (32.8%) patients, faded colour in three (4.7%) patients, difficulty reading in eight (12.5%) patients and vision fluctuations in 23 (35.9%) patients. The daily ethambutol dose was 15 mg/kg/d for all patients. The mean ethambutol treatment duration was 5.6 ± 4.0 months (range; 2–6 months) in the subclinical group, and 8.0 ± 2.9 months (range; 4–12 months) in the abnormal group.

Among the 126 eyes, 28 eyes in 14 patients (six female and eight male) were assigned abnormal visual function, i.e. decreased BCVA (< 0.1 by LogMar) in 15 eyes, visual field defect in 28 eyes, and dyschromatopsia in six eyes (two eyes with total dyschromatopsia and four eyes with red green dyschromatopsia) (Table 1). The remaining 98 eyes in the 50 patients (24 female and 26 male) had no visual dysfunction and were included in the subclinical group.

**Table 1:** Clinical presentation of the 14 patients with visual dysfunction

Case	Sex F/M	Age (y)	Duration Of EMB (mo)	BCVA R/L (LogMar)	Color test R/L	Optic Nerve(R/L)	Visual field Pattern(R/L)	Mean deviation (R/L,dB)
**1**	**M**	**72**	**7**	1.4/1.2	3 (11)/4 (11)	Pale/ Pale	CS/CS	−17.21/− 18.23
**2**	**M**	**62**	**9**	1.2/1.2	4 (11)/4 (11)	Normal/Normal	CS/CS	−12.34/−13.54
**3**	**M**	**54**	**8**	0.4/0.8	0 (11)/0 (11)	Normal/Normal	CCS/CCS	−13.15/−3.83
**4**	**F**	**23**	**4**	0.8/1.8	11 (11)/11 (11)	Normal/Normal	PC/PC	−12.33/−9.87
**5**	**M**	**67**	**6**	1.8/1.8	11 (11)/11 (11)	Normal/Normal	PC/PC	−4.68/−5.02
**6**	**M**	**30**	**8**	0.4/0.4	11 (11)/11 (11)	Normal/Normal	PC/PC	−6.88/−6.12
**7**	**F**	**74**	**10**	2.0/2.0	11 (11)/11 (11)	Normal/Normal	HA/HA	−6.67/− 6.23
**8**	**F**	**30**	**5**	0.1/0.2	11 (11)/11 (11	Normal/Normal	PC/PC	−5.32/−6.15
**9**	**M**	**67**	**10**	0.1/0.1	11 (11)/11 (11	Normal/Normal	CS/CS	−4.97/−5.23
**10**	**F**	**71**	**13**	0.1/0.1	11 (11)/11 (11)	Normal/Normal	AD/AD	−5.02/−4.99
**11**	**F**	**73**	**6**	0.1/0.1	11 (11)/11 (11)	Normal/Normal	HA/HA	−4.78/−4.12
**12**	**F**	**33**	**6**	0.1/0.1	11 (11)/11 (11)	Normal/Normal	HA/HA	−4.76/−4.12
**13**	**M**	**25**	**4**	0.0/0.0	11 (11)/11 (11)	Normal/Normal	CS/CS	**−5.23/−4.76**
**14**	**M**	**51**	**8**	0.1/0.1	11 (11)/11 (11)	Normal/Normal	HA/HA	−4.76/−4.12

*CCS* Cecocentral scotoma, *CS* Central scotoma, *PC* Peripheral constriction, *AD* Altitudinal defect, *HA* Hemianopsia

We observed no statistically significant differences between the three groups in terms of sex and age distribution. The Ethambutol treatment duration was statistically longer in the abnormal group than the subclinical group (*P* = 0.036) (Table 2). BCVA and MD in the subclinical group were similar to the control group, and there were significant differences in BCVA and MD between the abnormal and control group (Table 3).

**Table 2:** Comparison of basic clinical characteristics between subclinical, abnormal and control groups (mean ± standard deviation, *um*)

Subjects (eyes)	Control group 60 (118)	Subclinical group 50 (98)	Abnormal group 14 (28)	***P*** value^**a**^
**Sex (M/F eyes)**	**32/28**	**26/24**	**8/6**	**0.943**
**Age (y)**	**48.41 ± 15.86**	**45.05 ± 15.81**	**51.00 ± 21.02**	**0.187**
**EMB duration (mo)**	**–**	**5.6 ± 4.04**	**8.0 ± 2.94**	**0.036**^**b**^

Statistical significance was achieved at *P* < 0.05

*M* male, *F* female

**Table 3:** Comparison of visual functions between subclinical, abnormal and control groups. Median (IQR)

Subjects (eye)	Control group 60 (118)	Subclinical group 50 (98)	Abnormal goup 14 (28)	***P*** value^**a**^
**BCVA (LogMar)**	**0.00 (0.00–0.00)**	**0.00 (0.00–0.10)**	**0.95 (0.1–1.45)** ^**bc**^	**0.001**
**VFs (MD, dB)**	**−0.62(−1.29—0.35)**	**−0.98(−1.98–0.45)**	**−6.08(−11.48–4.98)**^**bc**^	**0.021**

*BCVA* Best corrected visual acuity, *VFs* Visual field sensitivity, *MD* Mean deviation

^a^Comparison among the three groups by 1-way analysis of variance

^b^Significant difference compared with the control group by post hoc multiple comparison

^c^Significant difference compared with the subclinical group post hoc multiple comparison

### OCT parameters for p-RNFL, GCIPL and total macular layer thickness

In contrast to the control group, p-RNFL thickness in the abnormal group increased significantly in the superior and inferior quadrants (GEE, *P* = 0.040, *P* = 0.010, respectively). In contrast to the subclinical group, p-RNFL thickness only in the inferior quadrant, increased significantly in the abnormal group (GEE, *P* = 0.047). We observed no significant difference in p-RNFL thickness between the subclinical and the control group (Table 4). In contrast to the control group, GCIPL thickness in inferonasal and inferior sectors decreased significantly in the subclinical group (GEE, *P* = 0.018, *P* = 0.047, respectively). In contrast to the control group, the average, and minimum value of GCIPL thickness together with superonasal, inferior, inferotemporal, superotemporal and superior sectors decreased significantly in the abnormal group (GEE, *P* = 0.016, *P* = 0.001, *P* = 0.028, *P* = 0.010, *P* = 0.012, *P* = 0.015 and *P* = 0.010, respectively). The average, and minimum value for GCIPL thickness, together with GCIPL thickness in inferior, inferotemporal, superotemporal and superior sectors were decreased significantly in the abnormal group when compared with the subclinical group (GEE, *P* = 0.023*, P* = 0.002*, P =* 0.048, *P =* 0.040 and *P* = 0.019, respectively) (Table 5).

**Table 4:** Comparison of p-RNFL thickness between the subclinical group, the abnormal group and the control group. (mean ± standard deviation, *um*)

Parameters Subjects (eyes)	Control group 60 (118)	Subclinical group 50 (98)	Abnormal group 14 (28)	***P*** value^**a**^
**Average**	108.00 ± 7.66	105.26 ± 8.89	112.83 ± 27.66	0.066
**Nasal**	75.38 ± 17.16	73.07 ± 10.61	77.37 ± 21.45	0.067
**Inferior**	138.61 ± 17.97	133.16 ± 16.63	151.88 ± 36.37^bc^	0.022
**Temporal**	84.74 ± 21.79	80.30 ± 14.19	81.17 ± 26.23	0.535
**Superior**	134.85 ± 17.83	130.60 ± 17.02	140.58 ± 36.97^b^	0.170

^a^Comparison among the three groups by generalized estimating equation

^b^Significant difference compared with the control group

^c^Significant difference compared with the subclinical group

**Table 5:** Comparison of GCIPL thickness between the subclinical, abnormal and control groups (mean ± standard deviation, *um*)

Parameters Subjects (eyes)	Control group 60 (118)	Subclinical group 50 (98)	Abnormal group 14 (28)	***P*** value^**a**^
Average	87.23 ± 4.26	82.93 ± 5.12	75.88 ± 7.36^bc^	0.034
Minimum	83.18 ± 4.58	79.17 ± 5.40	64.63 ± 14.22^bc^	0.003
Superonasal	90.41 ± 5.32	89.30 ± 5.98	77.29 ± 9.72^b^	0.070
Inferonasal	88.21 ± 5.25	83.29 ± 4.98^b^	76.29 ± 10.45	0.036
Inferior	84.38 ± 5.04	80.36 ± 5.43^b^	72.13 ± 8.28^bc^	0.015
Inferotemporal	86.26 ± 3.77	80.54 ± 5.40	77.08 ± 9.79^bc^	0.029
Superotemporal	86.36 ± 4.44	82.38 ± 5.91	76.46 ± 9.81^b^	0.040
Superior	88.23 ± 5.16	84.18 ± 5.82	76.71 ± 8.37^bc^	0.037

^a^Comparison among the three groups by generalized estimating equation

^b^Significant difference compared with the control group

^c^Significant difference compared with the subclinical group

For total macular retinal thickness measurements, we observed no significant differences in CST, CMV, or CAMT between the subclinical and control group. Only the CAMT in the abnormal group was significantly thinner than the control group (GEE, *P* = 0.027) (Table 6).

**Table 6:** Comparison of macular thickness between the subclinical, abnormal and control groups. (mean ± standard deviation, *um*)

Parameters Subjects (eyes)	Control group 60 (118)	Subclinical group 50 (98)	Abnormal group 14 (98)	*P* value^a^
CST (um)	242.95 ± 22.84	238.33 ± 23.07	231.67 ± 12.93	0.514
CMV (mm^3^)	10.23 ± 0.48	10.03 ± 0.49	9.77 ± 0.67	0.212
CAMT (um)	284.10 ± 13.96	277.97 ± 12.79	271.75 ± 18.12^b^	0.086

*CST* center subfield thickness, *CMV* Cube macular volume, *CAMT* Cube average macular thickness

^a^Comparison among the three groups by generalized estimating equation

^b^Significant difference compared with the control group

^c^Significant difference compared with the subclinical group

### Data from three EON patients

**Fig. 1:**
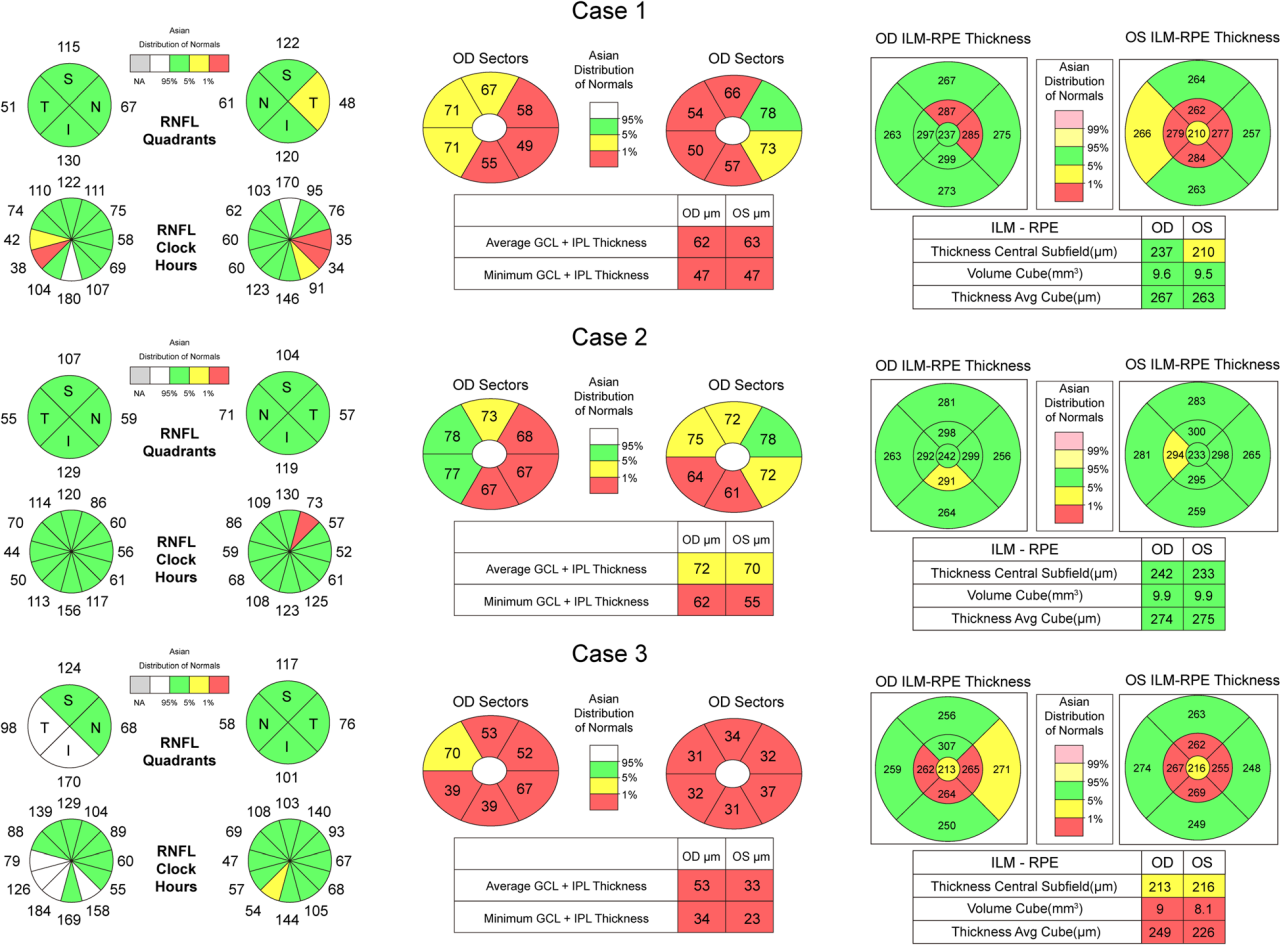
OCT scans of macular GCIPL, p-RNFL and total macular thickness in 3 EON cases. In case 1,the p- RNFL of temporal optic disc became thinner, and GCIPL thickness was significantly lower than normal primarly in nasal and nasal superior sectors which is associated with the papillo-macular bundls. In case 2, p-RNFL was normal in both eyes,but the changes in the thickness of GCIPL was similar to case 1. In case3, p-RNFL in the right eye increased,but the GCIPL of both eyes decreased apparently

Three patients in the abnormal group were diagnosed with EON. They complained of progressive visual decline after 7–9 months of ethambutol intake. Marked hypochromatopsia was detected using the 11-plate Ishihara Colour Test. One patient had a pale optic nerve head in both eyes, whereas the other two exhibited a normal appearance of the optic papilla (Table 1). The visual field test of all the three patients exhibited prominent defects, such as cecocentral and central scotomas. Of these patients, we observed different changes in the p-RNFL: one eye had slight p-RNFL thickening in the inferior quadrants, another eye was thinner in the temporal quadrant, and the remaining four eyes exhibited normal thickness. The GCIPL thickness in these EON patients was significantly thinner, especially in the nasal macular area (Fig. 1).

## Discussion

In this study, 14 patients had visual function damage after taking ethambutol. When compared with the control group, the p-RNFL, as measured by OCT, exhibited a significant thickening in superior and inferior quadrants.

Previous studies on p-RNFL thickness in EON patients indicated different conclusions depending on different EON stages [6–9]. In their follow-up of 31 subclinical EON patients, Kim et al. observed that the average p-RNFL was significantly thickened after 5 months of ethambutol intake, mainly at temporal and inferior quadrants. These authors considered that the increased p-RNFL thickness preceded the decrease in p-RNFL thickness, with the former triggered by axon swelling [5]. Chai et al. observed that temporal p-RNFL in their EON group was slightly thickened, but the difference was not significant when compared with the control group [6]. Zoumalant et al. reported that the p-RNFL had thickened in inferior quadrants, and swelling of the optic disc gradually decreased when the visual field gradually recovered after ethambutol was withdrawn [7], thus, they considered most cases were reversible when the p-RNFL was slightly thickened. In contrast, Chai et al. observed that the p-RNFL thickness had thinned in eight patients with EON; this thinning was most prominent in the temporal quadrant, and persisted for 12 months after the withdrawal of ethambutol [8]. In a prospective study by Menon et al.*,* three EON cases also exhibited a remarkable thinning of the temporal p-RNFL [9]. In a retrospective observational study in eight EON patients, Kim et al. observed that RNFL changes were not obvious at initial presentation, but layer thinning became obvious in two of their five patients over time [13].

Our data revealed that in early stage of EON, the p-RNFL may appear as a slight swelling, with thickening often occurring in the inferior and superior quadrants of the optic papilla. These areas are related to the aggregation of large diameter M-cell axons, which are prone to swelling. The temporal p-RNFL corresponding to the papilla macular bundles is conposed of small P-cell axons, whose smaller volume to surface area ratio makes them have the characteristics of high-energy demand and low-energy storage. When the axon lacks energy, the papilla macular bundle is inclined to be damaged [14]. In our study, 14 patients had a short course of onset within 6 months, and three were diagnosed with EON. The remaining 11 did not completely meet EON diagnosis criteria, and may have been in early stages; their papilla macular bundle fibres were partially compensated and swollen, whereas others were atrophied, which potentially resulted in no significant differences in the thickness of the temporal nerve fibre layer when compared with the control group.

Peripapillary-RNFL thickness is affected by several factors [8, 9]. Even in normal populations, p-RNFL thickness varies considerably, but ganglion cell distribution in the macular area is relatively stable. Our data indicated that GCIPL was significantly thinned in both study groups after ethambutol administration, but the degree of thinning was significantly higher in the abnormal than the subclinical group. Some studies have suggested that while ethambutol exhibits neurophilic characteristics, it may also cause primary damage to retinal ganglion cells [10]. Peng et al. measured total macular thickness by OCT, and observed that the inner circle and outer nasal sectors containing primarily papilla macular bundles were thinner in EON-affected eyes when compared with healthy controls [15]. Han et al. found no abnormal p-RNFL and GCIPL parameters in 36 patients who had no abnormal visual functions within 6 months after taking ethambutol. However, in one patient diagnosed with EON after taking ethambutol, the p-RNFL was thickened, whereas the GCIPL was thinned [16]. Lee et al. reported that the degree of GCIPL thinning was closely related to an EON prognosis [17]. Vieira et al. showed that the GCIPL in patients with EON became thinner in each quadrant, and was positively related to defects in MD [18]. Teng et al. also found that the GCIPL was significantly thinner in patients with EON, and suggested that whether the p-RNFL was swollen or atrophic, that loss of ganglion cells in the macular region had occurred [19].

Our study data were consistent with previous reports on GCIPL thickness [16–19]. After taking ethambutol for > 2 months, our patients complained with various degrees of visual impairment. Those patients with decreased visual function experienced GCIPL thinning. Patients in the subclinical group, with normal visual function, also exhibited significant differences in GCIPL thinning when compared with controls, whereas the p-RNFL thickness was similar to controls. These findings suggested that retinal ganglion cells may be the initial site of ethambutol damage. Apoptosis of retinal ganglion cells could occur before visual dysfunction. The p-RNFL in EON patients may be slightly swollen or exhibit no characteristic changes in disease stages; however, the GCIPL generally becomes thinner. In the abnormal group, we noted that CAMT thickness, which was the average macular fovea thickness within the diameter of a 1 mm circle, was significantly thinner than the control group.

Our study outcomes suggest that GCIPL thickness measurements using Cirrus-HD OCT may be more sensitive than p-RNFL thickness. However, as this was a retrospective study, and we accounted for differences between individuals and statistical methods, we were unable to confirm the utility of GCIPL as a more sensitive and reliable parameter in screening early EON disease. A longitudinal study with a larger sample will help clarify damage to retinal ganglion cells caused by ethambutol, and allow clinicians to recognise the damage caused at early EON stages.

## Conclusions

GCIPL measurements using Cirrus-HD OCT detects retinal ganglion cell layer loss following ethambutol treatment, before visual dysfunction. Using p-RNFL and GCIPL thickness parameters, Cirrus-HD OCT helped detect damage patterns in retinal ganglion cells caused by ethambutol, and may indicate ethambutol withdrawal before serious visual impairment occurs.

## Data Availability

The datasets during the current study are available from the corresponding author on reasonable request.

## Abbreviations

Cirrus-HD OCT: Cirrus high- definition optical coherence tomography
p-RNFL: Peripapillary retinal nerve fiber layer
GCIPL: Ganglion cell-inner plexiform layer
EON: Ethambutol-induced optic neuropathy
CST: Center subfield thickness
CMV: Cube macular volume
CAMT: Cube average macular thickness
BCVA: Best-corrected visual acuity
GEE: Generalised estimating eq.
MD: Mean deviation
